# Supplementary material for: Ultrafast 3D nanofabrication via digital holography
Source: Nat Commun. 2023 Mar 27;14:1716. doi: 10.1038/s41467-023-37163-y (PMC10043265; doi:10.1038/s41467-023-37163-y)
Supplement: Supplementary file 1 — Supplementary information [file 41467_2023_37163_MOESM1_ESM.pdf]

Supplementary Information

Ultrafast 3D Nanofabrication via Digital Holography

Wenqi Ouyang[1], Xiayi Xu[1,2], Wanping Lu[1], Ni Zhao[2,3], Fei Han[1,2,*], and Shih-Chi Chen[1,2,4,*]

Affiliations:

[1]Department of Mechanical and Automation Engineering, The Chinese University of Hong Kong, Shatin, N.T., Hong Kong.

[2]Hong Kong Centre for Cerebro-Cardiovascular Health Engineering (CoCHE), Shatin, N.T., Hong Kong.

[3]Department of Electronic Engineering, The Chinese University of Hong Kong, Shatin, N.T., Hong Kong.

[4]Centre for Perceptual and Interactive Intelligence (CPII), Shatin, N.T., Hong Kong.

*Corresponding author. Email: scchen@mae.cuhk.edu.hk (S.-C.C.); feihan@cuhk.edu.hk (F. H.);

This PDF file includes

Supplementary Methods
Supplementary Table 1-4
Supplementary Figures 1-17
References

Supplementary Methods

System configuration

A detailed optical setup of the multi-focus TPL printing system is presented in Supplementary Fig. 1. To provide sufficient power for more than 2000 foci to simultaneously induce two-photon polymerization, a Ti:sapphire regenerative laser amplifier system (Spitfire Pro, Spectra-Physics; central wavelength: 800 nm; repetition rate: 1 kHz; pulse width: 100 fs; average power: 4 W) is selected as the light source. A half wave plate (HWP) and a polarizing beam splitter (PBS) are used to adjust the laser power. To generate the scanning laser foci, a DMD (DLP 6500, 1920×1080 pixels, pixel size: 7.56 μm, Texas Instrument), which is synchronized to the laser pulses at 1 kHz, displays the designed binary holograms. After the DMD, the diffracted laser beam generates the laser foci at the Fourier plane of L3 (f_{L3} = 150 mm), where a spatial filter is used to block redundant diffraction orders. Owing to the small DMD pixel size, angular dispersion can be introduced, causing unwanted pulse broadening effect. To address this issue, a dispersion pre-compensation unit is designed and placed before the DMD, which includes a reflective blazed grating (600 lines/mm) and two lenses (L1 and L2; f_{L1} = 225 mm, f_{L2} = 250 mm) that form a 4-f system to adjust the beam size to fully fill the DMD aperture. Lastly, a second 4-f system (L4 and L5, i.e., objective lens) relays and rescales the laser foci from the Fourier plane of L3 to the sample plane for nanofabrication. To monitor the fabrication process in situ, a trans-illumination microscope is built in conjunction with the fabrication system, which shares the objective lens. An LED light source (GCI-060402, Daheng Optics; central wavelength: 589 nm) is coupled to the system via a single mode fiber and a mirror (M1) below the glass substrate. Next, the transmitted light (image) passes through the objective, a dichromic mirror (DM) and a lens (L6) to form an image on the CCD camera (BFLY-U3-26S6C-C Blackfly, FLIR). Notably, the objective and L6 form a 4-f system to appropriately magnify the image. The glass substrate is placed on a precision six-axis positioner (H-811.I2, Physik Instrumente), which helps to minimize stitching errors for printing large parts and eliminate substrate tip-tilt errors. A high numerical aperture (NA) oil immersion objective lens (Nikon CFI S Fluor 40× Oil, NA = 1.3, WD = 0.24 mm) is used to match the refractive index of the photoresist and improve fabrication resolution. The power efficiency of the grating, DMD, and the objective lens is measured to be 64%, ~5% and 82% respectively. The overall efficiency of the system is ~2.62%.

Compensation of angular dispersion introduced by the DMD

This section presents the design of the dispersion pre-compensation unit, which consists of a reflective blazed grating and a 4-f system (L1 and L2; f_{L1} = 250 mm, f_{L2} = 225 mm). First, the grating equations, i.e., Eq. (1) and (2), are used to calculate the parameters for the unit, where the diffraction order of the grating is m_G = 1; the diffraction order of the DMD is m_D = 3; the line width of the grating is d_G = 1/600 mm; the line width of the DMD is d_D = 7.56/√2 μm, and the wavelength is λ = 800 nm. As illustrated in Supplementary Fig. 2a, θ_{iG}, θ_{mG}, θ_{iD}, and θ_{mD} which respectively denote the laser incident angle and diffraction angle of the grating and DMD in reference to the surface normal vector. In addition, $\theta_G = \theta_{iG} - \theta_{mG}$ and $\theta_D = \theta_{iD} - \theta_{mD}$.

$$\mathrm{m}_G\lambda = d_G(\sin(\theta_{iG}) + \sin(\theta_{mG})) \quad (1)$$

$$\mathrm{m}_D\lambda = d_D(\sin(\theta_{iD}) + \sin(\theta_{mD})) \quad (2)$$

To fulfil the requirement of chromatic compensation, the diffraction angle of the grating (θ_{mG}) and the incident angle of the DMD (θ_{iDG}) should follow the relation expressed in Eq. (3):

$$\frac{d\theta_{mG}}{d\theta_{iD}} = f_{L2}/f_{L1} \quad (3)$$

Thus, by differentiating Eq. (1) and (2) and combining the results with Eq. (3), the relation between the grating diffraction angle and the DMD incident angle can be derived as:

$$\frac{d_D \cos(\theta_{iD})}{m_D f_{L2}} = \frac{d_G \cos(\theta_{mG})}{m_G f_{L1}} \quad (4)$$

Based on Eq. (1) - Eq. (4), the values of θ_G, θ_{mG}, θ_D, θ_{iD} and θ_{mD} are plotted as a function of θ_{iG} in Supplementary Fig. 2 (b). To fully compensate the angular dispersion, θ_{iG} should be larger than 48.80°. Based on the analysis, the parameters of the dispersion pre-compensation unit are set as follows: θ_{iG} = 49.43°, θ_{mG} = -16.24°, θ_{iD} = 3.80°, θ_{mD} = 22.50° and θ_D = 18.70°.

System Parameters

The detailed parameters of multi-focus TPL system are provided below:

- Scanning range: 299 µm, 554 µm, and 760 µm in the *x*, *y*, and *z* direction
- Scanning resolution: 128 nm in *x*, 128 nm in *y*, and 249 nm in *z*
- Minimum distance between two arbitrary foci: 3 µm
- Scanning rate (i.e., laser repetition rate): 1 kHz
- Pulse energy per focus to achieve the best results: 5 nJ
- Laser peak power density per focus: 15 - 70 TW/cm^2
- Number of foci: 1 - 2000

Typical processing parameters are presented in Table S1 to compare our multi-focus TPL printing system with the conventional point-scanning TPP process.

Estimation of fabrication rate based on voxels vs. volume

The voxel fabrication rate is defined by the product of the number of laser focus (*N*) and the laser repetition rate (1 kHz):

$$v_{voxel} = N \times 1000\, voxels/s \quad (5)$$

In our system, the maximum number of scanning foci to achieve quality results is 2000; accordingly, the voxel fabrication rate (v_{voxel}) is 2,000,000 voxels/s. Considering most functional microstructures have a volume-filling ratio (η) of 1% -12% (for lattice structures)[1,2] and 10 - 60% (for shell structures)[3,4], we can convert the voxel fabrication to the volume fabrication rate (v_{volume}) by Eq. (5), where the lateral and vertical distances between two nearby voxels are set to be 300 and 1000 nm, respectively[5]. Based on this, the volume fabrication rate for our system is 5.4 - 64.8 mm^3/hour, corresponding to 1% - 12% volume-filling ratio. Based on our experience, if stitching is needed (for fabricating large structures), the total fabrication time will be increased by ~20%.

Accordingly, the volume fabrication rates for large structures are estimated to be 4.5 - 54.0 mm^3/hour.

$$v_{\text{volume}} = \frac{N \times \text{voxel volume}}{\mu \times \text{voxel printing time}} = \frac{N \times 3.24 \times 10^{-4}}{\mu} \ (\text{mm}^3/\text{hour}) \tag{6}$$

To fairly compare the fabrication rate, we report our fabrication in both formats, i.e., voxel printing speed and volume fabrication rate, at different volume-filling ratios (see Fig. 3a). Supplementary Table 2 presents the volume fabrication rate of the designed meta-structures (volume-filling ratio: 10-20%) under different conditions and parameters.

Synthesis of binary holograms for multi-focus random-access scanning

Fast wavefront control of a dispersion-free femtosecond laser beam is achieved by applying binary holography (i.e., Lee holography) to a DMD[6,7]. Eq. (7) presents the governing equation of binary holography. To implement it on a DMD, let $A(x,y) \cdot \exp[i\varphi(x,y)]$ be the target wavefront, where $A(x,y) \in [0,1]$ and $\varphi(x,y)$ represent the amplitude and phase of the electric field respectively; x and y are the coordinates in the Cartesian coordinate system. Next, let $h(i,j) \in \{0,1\}$, $(1 \le i \le m, 1 \le j \le n, i, j \in N)$ be the pixels on the DMD, where 1 and 0 refer to the "on" and "off" states respectively; and m and n refer to the number of rows and columns respectively, i.e., $x = i \cdot d_D$ and $y = j \cdot d_D$.

$$h(i,j) = \begin{cases} 1, & -\frac{\sin^{-1} A(x,y)}{2\pi} \le \frac{R(x,y)}{T} + \frac{\varphi(x,y)}{2\pi} + k \le \frac{\sin^{-1} A(x,y)}{2\pi} \\ 0, & otherwise \end{cases}, \tag{7}$$

Single-focus random-access scanning can be achieved by superposing lateral and axial scanning holograms and rapidly modulating them in the DMD. Firstly, lateral scanning is realized by modulating the tilted phase term, $R(x,y)$, in Eq. (7), which controls the spatial separation of different diffraction orders; T is the grating period and k is an integer. Generally, the tilted phase is given by $R(x,y) = \cos\theta \cdot x + \sin\theta \cdot y$, where θ is the angle of the fringes measured counter-clockwise from the y-axis. Accordingly, by controlling T and θ, the first diffraction order can be shifted laterally[8]. Secondly, axial scanning is realized by applying binary holograms of spherical wavefronts of increasing or decreasing focal lengths to the DMD. The spherical wavefronts are mathematically expressed in Eq. (8)[9] below:

$$\varphi(x,y) = \frac{\pi(x^2 + y^2)}{\lambda f}, \tag{8}$$

The generation of multiple laser foci can be achieved by superposing designed binary holograms, where each constituent hologram contains the spatial and intensity information of a designed laser focus. This is feasible as the target wavefront $A(x,y) \cdot \exp[i\varphi(x,y)]$ can contain many frequency components instead of one. As such, all focal points generated by the hologram can be independently controlled and arbitrarily positioned in the same work space. Through the intensity distribution control among the focal points, single exposure grayscale control is achieved. To express multi-focus generation mathematically, let $A(x,y) \cdot \exp[i\varphi(x,y)]$ be the target wavefront

containing k focal points. Binary holograms with the desired intensity distribution among the k focal points can be synthesized via Eq. (9),

$$h(i,j) = \begin{cases} 1, & -A(x,y) \le \sum_{k=1}^{n} B_k \sin\left(2\pi \frac{R_k(x,y)}{T_k} + \varphi_k(x,y) + \varphi_{w,k}(x,y)\right) \le A(x,y) \\ 0, & otherwise \end{cases}, \quad (9)$$

where $h(i, j)$ represents the binary value of the micromirrors on the DMD; B_k, $R_k(x,y)$, T_k and ϕ_k are the relative amplitude factor, tilted phase, grating period, and phase for the kth focal point respectively. $\phi_{w,k}$ is the additional wavefront information to be included in the hologram for controlling the size and shape of the focal points. Note that the power of each focus can be individually controlled by adjusting B_k. In practical applications, due to the limited total DMD pixels, increasing the number of laser foci may slightly compromise the printing resolution. Lastly, multi-focus random-access scanning is realized by switching the designed holograms in the DMD memory, where each hologram contains the amplitude, phase, and location information of the designed laser foci. Video demonstrations are presented in the Supplementary Video 1 and 2 in 20× slow motion for better visualization.

The relationship between the scanning performance, i.e., range and resolution, and the DMD parameters, i.e., pixel size (d_D) and aperture sizes ($\sim nd_D$), has been studied in our previous work[8-10]. The results are applicable to both single-focus and multi-focus scanning processes. Briefly, for lateral scanning, the range is inversely proportional to d_D and the magnification of the objective lens (M_{obj}); the minimum step size is inversely proportional to nd_D and M_{obj}. For axial scanning, the range is inversely proportional to d^2 and M_{obj}^2; the minimum step size is inversely proportional to n^2d^2 and M_{obj}^2. When using a 40x oil objective lens, the work volume of the DMD scanner is calculated to be 299 × 554 × 760 μm^3; and the scanning resolution is 128 nm and 249 nm in the lateral and axial directions respectively.

Intensity distribution among laser foci

Highly uniform intensity distribution among the generated scanning laser foci is critical to the fabrication of complex metastructures. Yet, generation of a multi-focus hologram by direct superposition, i.e., Eq. (9), can result in non-uniform intensity distribution. The effect becomes increasingly prominant as the number of foci increases. We address this issue by applying an iterative optimization algorithm, i.e., weighted Gerchberg–Saxton (WGS) algorithm, to the designed digital holograms, which ensures the intensity uniformity can reach 99% for up to 1000 laser foci. In addition, for grayscale writing, where each focus carries different laser power, the WGS algorithm is also applied to improve the accuracy of the designed holograms.

The WGS algorithm effectively optimizes the uniformity and accuracy of the laser power through iterative calculation, as expressed in Eq. (10). To begin, two coefficients, w_t and θ_t, are introduced to adjust the weight and phase of the tth focus; l is the number of iteration; ϕ_t is the phase of the tth focus. After each iteration, the phase ϕ^l is updated by Eq. (10). The weight of the amplitude and the phase is iterated according to Eq. (11) and Eq. (12), where V_t is the normalized electric field intensity, calculated by Eq. (13); I_t^d is the desired intensity of the tth focus. If a uniform intensity distribution is desired, I_t^d is set to be equal for all foci. For grayscale control, different values of I_t^d will be assigned to each focus. To examine the result by the lth iteration, the overall

uniformity u^l is calculated, as expressed in Eq. (14), where $I_{max}{}^l$ and $I_{min}{}^l$ are the maximum and minimum value of the normalized intensity of each focus $I_t{}^l$, as defined in Eq. (15).

$$\phi^l(x,y) = arg\left[\sum_t w_t^l\, e^{i(\phi_t(x,y)+\theta_t^l)}\right], \tag{10}$$

$$w_t^{l+1} = w_t^l \frac{\langle |V_t| \rangle}{|V_t|} I_t^d, \tag{11}$$

$$\theta_t^{l+1} = arg\left(V_t^l\right), \tag{12}$$

$$V_t^l = \sum_{i=1}^{m} \sum_{j=1}^{n} H^l(i,j) e^{i(-\phi_t(x,y))}, \tag{13}$$

$$u^l = 1 - \frac{I_{max}^l - I_{min}^l}{I_{max}^l + I_{min}^l} \tag{14}$$

$$I_t^l = \left|V_t^l\right| / I_t^d \tag{15}$$

To achieve uniform intensity distribution, we start the iteration process by assigning equal intensities and random phases to Eq. (10), i.e., $w_t = 1$, and $\theta_t \in (0, 2\pi)$. Depending on the number of laser focus, e.g., from 9 – 1000 foci, the algorithm will converge to better than 99% uniformity within 1 – 80 iterations. Supplementary Fig. 10c presents the optimal uniformity as a function of number of laser focus and iterations. Note that in the simulation we assume the minimal focus distance is larger than 3 μm to avoide focus merging or bulk polymerization (See next section for more discussion). From the results, it is found that for 9, 100, 400, 1000 and 4000 foci, the optimal uniformity is calculated to be 99.98%, 99.89%, 99.67%, 99.48%, and 98.57%, respectively.

Proximity effect and solution

Over-polymerization can occur when 20 or more laser foci are simultaneously employed, which results in unwanted effects such as expanded linewidths or bulk solidification, as shown in Supplementary Fig. 8c. This is mainly attributed to (1) the close proximity of the scanning foci and the diffusion of the nearby reaction species, which together cause laser doses to accumulate in a specific region; and (2) non-uniform laser power distribution in the diffraction envelope, as illustrated in Fig. 6a and b, which overlaps with the DMD work volume. To compensate the non-uniform laser power in the diffraction envelope, we have included the background laser power in the WGS algorithm such that the resulting laser power for each focus achieves high uniformity with the presence of the diffraction envelope. To avoid the focus proximity effect, a minimum distance between any two scanning foci can be set. For example, if a minimum distance of 3 μm is set, up to 2000 laser foci can operate simultaneously without causing the proximity effect, as demonstrated in Supplementary Figure 9.

Actuation of the magnetic micro-gear

The micro-gears were removed from the substrate after printing and development. Supplementary Fig. 17 presents the optical image of an array of micro-gears on the glass substrate after development. The actuation of the micro-gear was performed in an aqueous environment (PVP, 6% wt., Aladdin) and a PMMA chamber. The programmable magnetic field was generated by custom-built Helmholtz coils with a magnetic field strength of 10 mT. Based on the setup, we demonstrate two modes of gear motion. In Supplementary Video 3, the micro-gear rotated clockwise at 5 Hz, which confirms good magnetic property has been achieved via our photoresin. In Supplementary Video 4, the micro-gear moved along the design trajectory with a flipping motion at 1 Hz, which demonstrates good maneuverability for robotic applications.

Supplementary Tables

Supplementary Table 1. Comparison of key printing parameters between the multi-focus TPL fabrication system vs. conventional point-scanning system.

	Multi-focus TPL system	Point-scanning system
Laser repetition rate	1 kHz	80 MHz
Intensity under objective lens (for a single focus)	5 μW	8 mW
Laser peak power density (for a single focus)	26.2 TW/cm^2	0.52 TW/cm^2
Exposure time	100 fs	0.2 ms
Number of pulses to define (polymerize) a voxel	1	16,000
Pulse energy	5 nJ	1.6 μJ
Peak power (for a single focus)	50,000 W	100 W

Supplementary Table 2. Volume filling ratio and volume fabrication rate (v_{volume}) of the designed metastructures in Fig. 4 and Supplementary Fig. 12 of different unit sizes, fabrication time, volume, and number of printing foci.

Number of foci	Unit size (μm^3)	Total volume (μm^3)	Total fabrication time (s)	Volume filling ratio (%)	Volume fabrication rate (mm^3/hour)	Related figure
64	8 × 8 × 13.5	90 × 90 × 81	1.51	1.33	1.56	Fig. 4a
100	8 × 8 × 13.5	112 × 112 × 108	2.02	1.34	2.42	Fig. 4b
400	5 × 5 × 8	141 × 141 × 112	2.35	3.80	3.41	Fig. 4c
1024	4 ×4 × 6	180 × 180 × 30	0.70	6.64	5.00	Supplementary Fig. 12a

Supplementary Table 3. Printing speed and resolution of the works cited in Fig. 3.

Serial number	Reference number in the article	Volume fabrication rate (v_{volume}) (mm^3/hour)	Voxel fabrication rate (v_{voxel}) (voxels/s)	Lateral resolution (nm)
S1	25	20*	~333,000,000	140
S2	21	7.7×10^{-5}	300	454
S3	22	0.024	10,000	500
S4	20	1.15	9,000,000	400
S5	19	3.63×10^{-4}	1135	250
S6	41	5×10^{-5}	4,500	120
S7	42	5×10^{-4}	40,000	100
S8	43	0.013	300	1,000
S9	44	0.25	160	2,000
S10	45	2.76×10^{-3}	3,000	500
**This work**	**-**	**4.5 - 54.0**	**1,000,000**	**90**

*Volume fabrication rate for S1 is calculated based on a voxel axial distance of 4 µm.

Supplementary Table 4. Measured polymerization threshold, damage (explosion) threshold, and the dynamic range of selected photoresists for our TPL system. The parameters are found based on single-focus operation with single pulse exposure. (See Supplementary Fig. 4 for more characterizations.)

Type of photo-initiator	DETC (3% wt.)	IP-DIP	CAS 55035-43-3 (0.4% wt.) (Photoresist used in this work)
Polymerization threshold, E_1	5.20 nJ	7.90 nJ	1.27 nJ
Damage threshold, E_2	16.10 nJ	26.50 nJ	17.10 nJ
Dynamic range, $(E_2 - E_1) / E_1$	2.12	2.38	12.46

Supplementary Figures

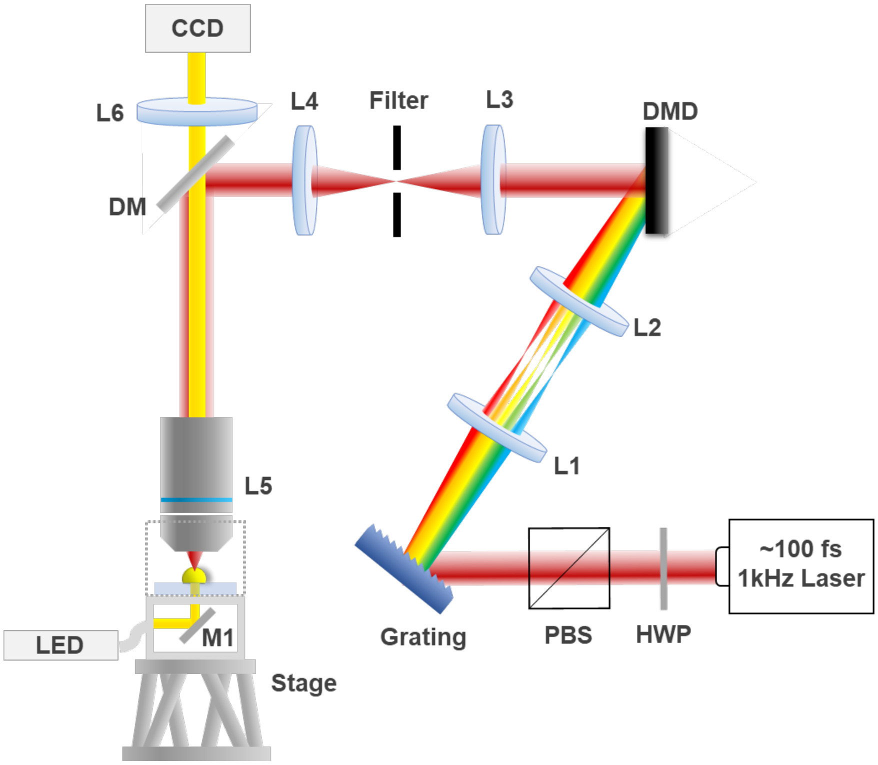

Supplementary Figure 1. Optical configuration of the multi-focus TPL system. HWP, half-wave plate; PBS, polarizing beam splitter; DM, dichroic mirror; L1-L4 and L6: lenses (f_{L1}, f_{L2}, f_{L3}, f_{L4}, f_{L6} = 225, 250, 150, 200, and 100 mm, respectively); L5: Objective lens; M1: Mirror.

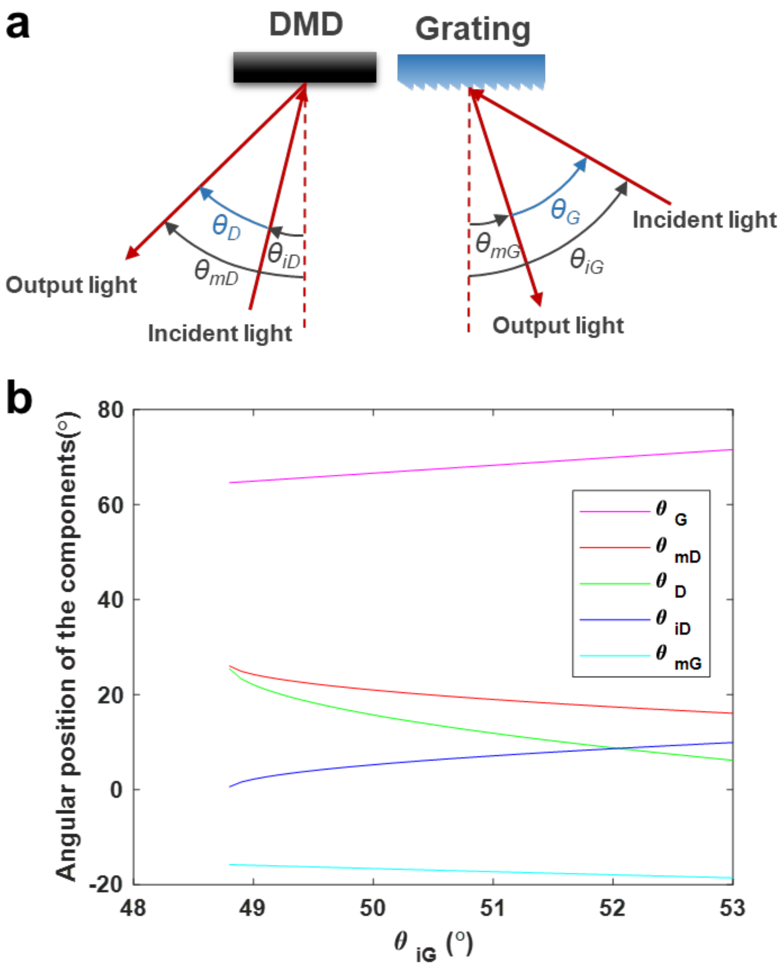

Supplementary Figure 2. (**a**) Definition of θ_{iG}, θ_{mG}, θ_G, θ_{iD}, θ_{mD}, and θ_{iD}. (**b**) θ_G, θ_{mG}, θ_{iD}, θ_{mD}, and θ_{iD} plotted as a function of θ_{iG}.

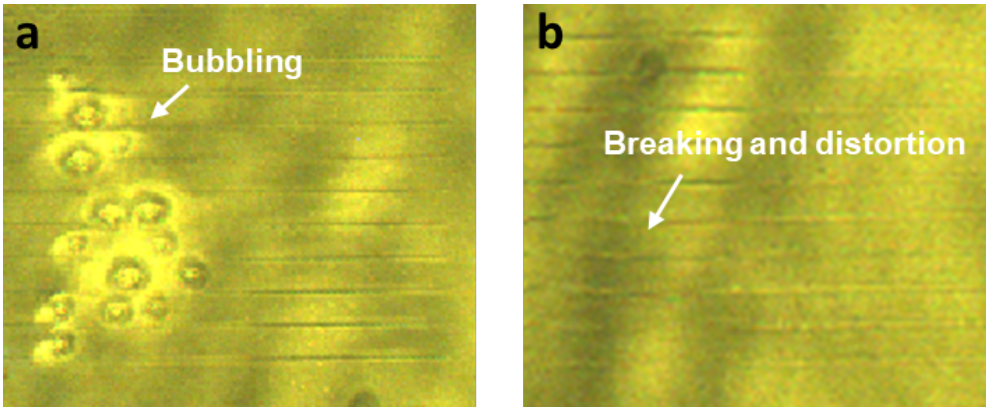

Supplementary Figure 3. Nanofabrication via the multi-focus TPL system: (**a**) in situ optical image showing micro-explosions during laser exposure in IP-DIP^{TM}; and (**b**) the resulting disconnected lines after the bubbles disappear.

Supplementary Note 1: Owing to the high polymerization threshold (7.9 nJ) and narrow dynamic range (i.e., 2.4) of IP-Dip^{TM} (Nanoscribe GmbH) in our multi-focus TPL system, over-polyermization and micro-explosions can occur when the laser pulse energy increases to above the threshold energy, making process control difficult. In the above image, a measured pulse energy of 15 nJ (under the objective lens) was applied to perform single-shot fabrication, which caused overpolermization and distortion of the printed structures, as shown in Supplementary Fig. 3a and 3b, respectively. After investigation, we found the unexpected increase in pulse energy was attributed to the back reflection in glass substrate[11]. Custom-designed photoresists of high dynamic range can largely address the issue and simultaneously improve the reproducibility when printing sub-diffraction limit structures.

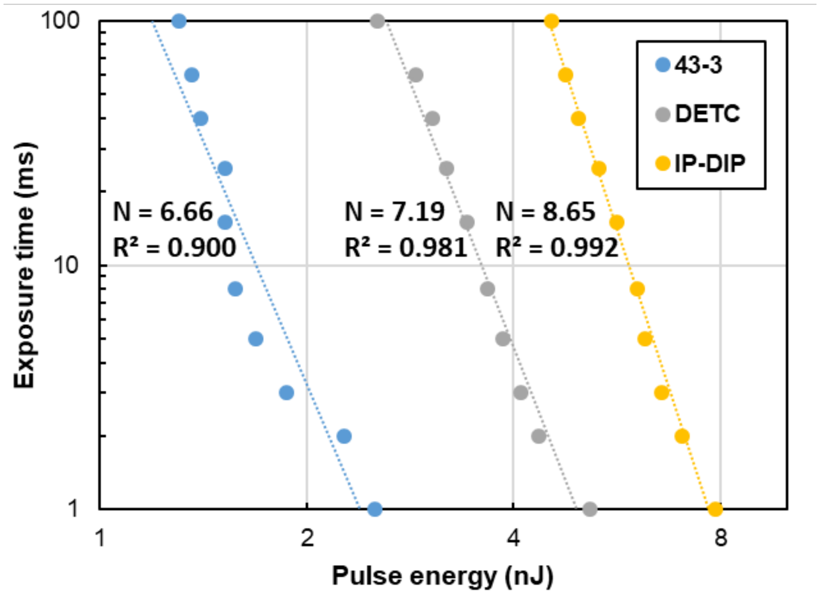

Supplementary Figure 4. Study of polymerization threshold using different combinations of exposure time (i.e., number of laser pulses) and pulse energies on different photoresists, including IP-DIP^{TM}, custom-developed photoresists based on DETC and CAS 55035-43-3 (shown in Fig. 2a). The data are used to calculate the non-linear coefficient (*N*) of the photoresists.

Supplementary Note 2: We measured polymerization threshold using different combinations of exposure time (i.e., number of pulses) and pulse energy to calculate the non-linear coefficient (*N*) of the photoresist via the equation $D \propto \tau R {E_\mathrm{p}}^N$, where D is the laser dose; τ is the exposure time; R is the laser repetition rate; and E_p is the pulse energy[12]. From this equation, the logarithmic value of the pulse energy and the exposure time have a linear relation as $log\ (\tau) = -N \log(E_p) + C$, where C is a constant. Next, N is experimentally determined by linearly fitting the measured experimental data in the above figure. In the experiments, we tested three photoresists, including IP-Dip^{TM} and custom-developed photoresists using two different photo-initiators (3% wt. of DETC and 0.4% wt. of chemical CAS 55035-43-3); other photoresists components are described in the main paper). The calculated nonlinear coefficients are 8.65, 7.19, and 6.65 for the three resins, repectively. This suggests that multiple photons have participated in the photopolymerization process induced by the ultrahigh peak power laser. Owing to the higher two-photon cross-section of CAS 55035-43-3, it may promote the probability of 2PA, resulting in improved fabrication characteristics (i.e., low polymerization threshold and high dynamic ranges, see Supplementary Table 4 for details) and results. (Note that *N* should be close to 2-3 for 2PA processes; *N* may vary slightly depending on the types of the photoinitiators.) Lastly, based on Supplementary Fig. 4, suitable pulse energy and laser doses can be determined for a nanofabrication process.

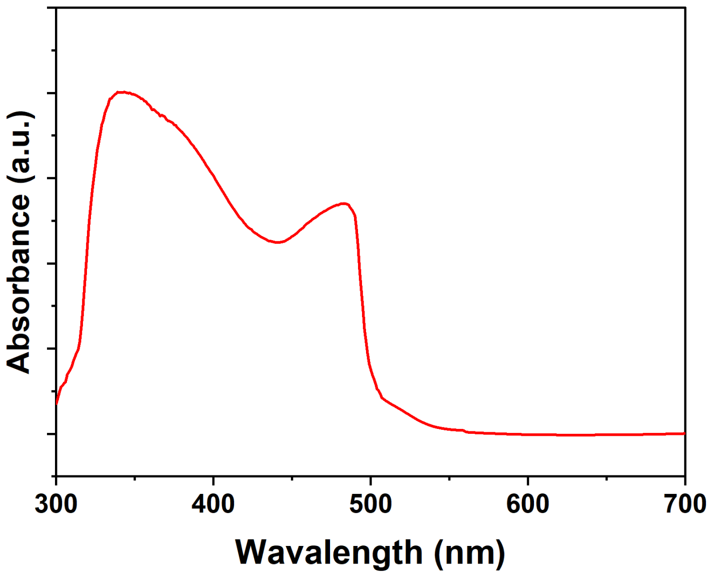

Supplementary Figure 5. Ultraviolet-visible absorption spectrum of the custom-designed photoresist.

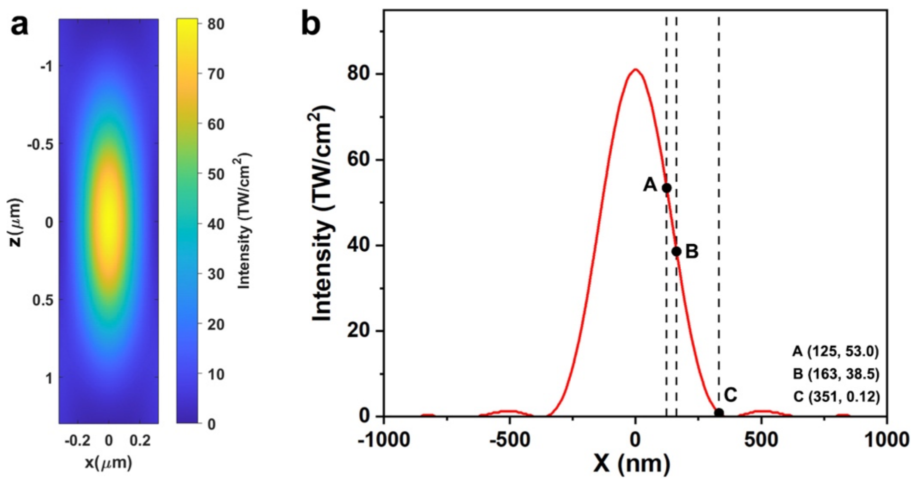

Supplementary Figure 6. (a) Simulated intensity distribution at the focal spot using the printing parameters in Fig. 2i (pulse energy = 4.5 nJ; repeition rate = 1 kHz; pulse width = 100 fs); (b) Cross-sectional diagram of (a) at *z* = 0 along the lateral direction. Point A is the polymerization boundary of the first pulse; point B is the final polymerization boundary (i.e., size) of the 1st voxel; and point C represents the limiting radius with the influence of diffusion. The simulation was performed based on the scalar-based point-spread function model in the literature[13], where the refractive index was set to 1.52; and the NA of the objective lens was 1.3.

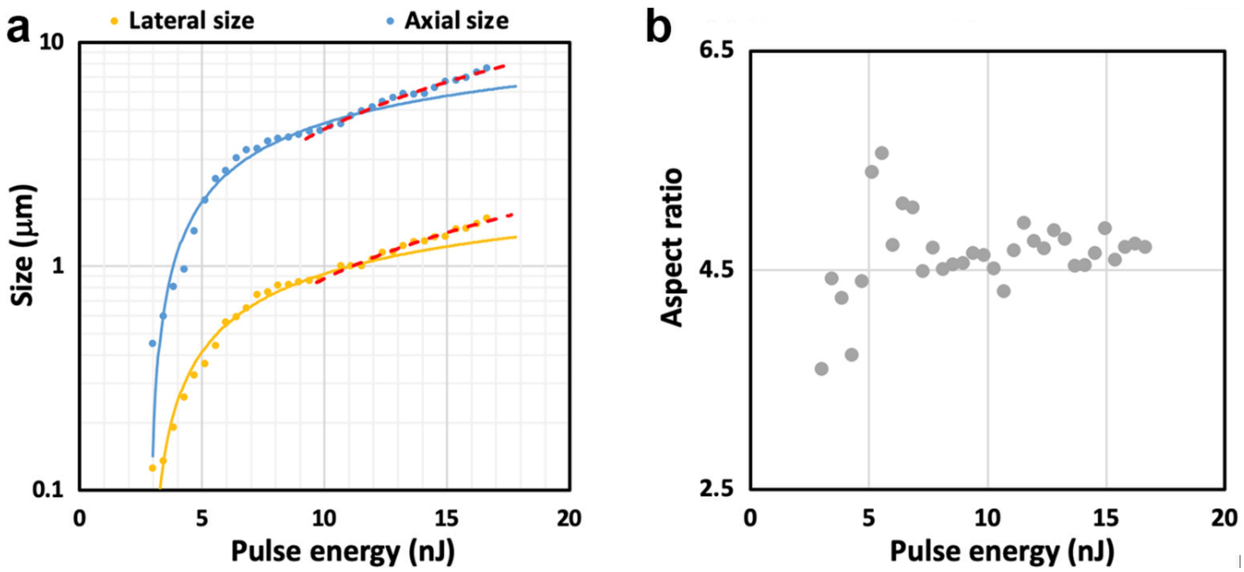

Supplementary Figure 7. Experimental results that show the evolution of (a) linewidth, and (b) voxel aspect ratio with increasing pulse energy from 3 to 18 nJ in an array of polymer nanowires printed via single-pulse exposure processes. The trendlines were generated via logarithmic function and linear function fitting, where one can observe an abrupt change of the direction of trendlines at a pulse energy of ~10 nJ.

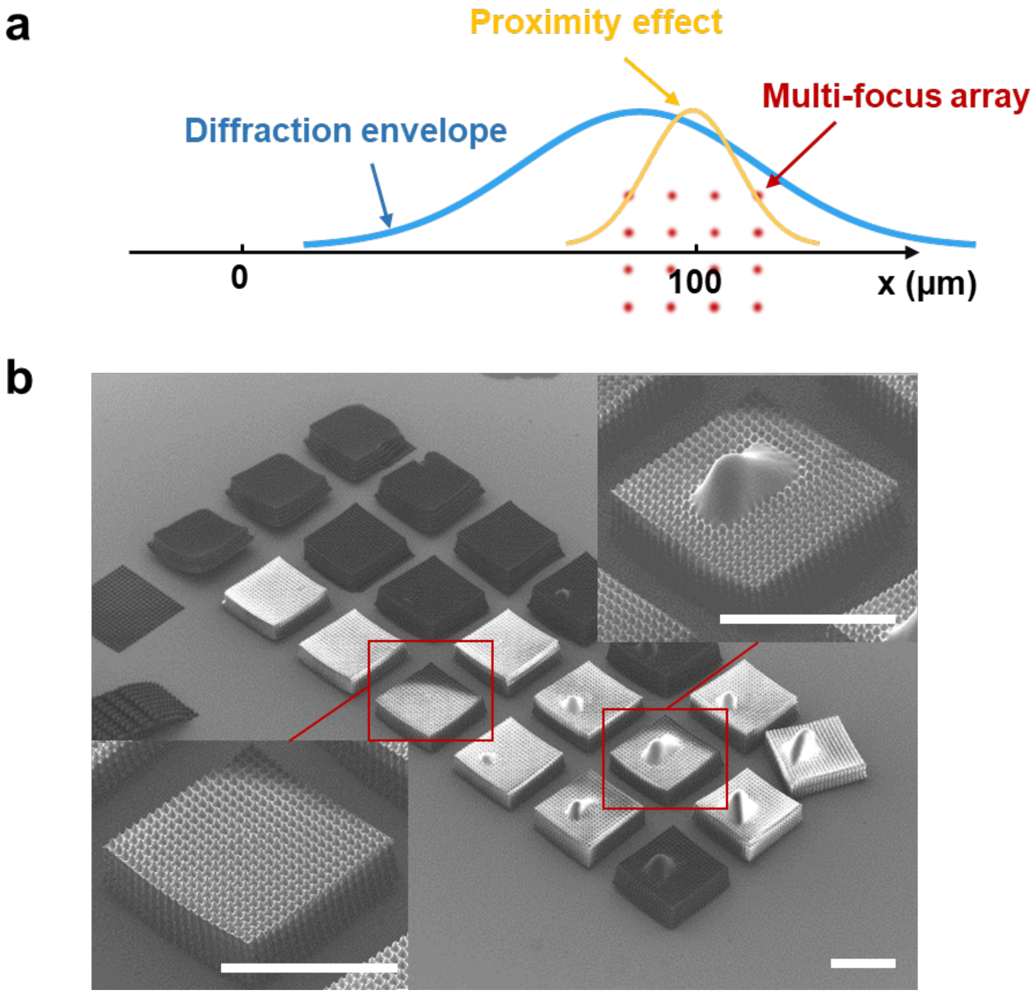

Supplementary Figure 8. (**a**) Schematics showing the diffraction envelope and the focus proximity effect, which causes laser power to accumulate and accordingly results in bulk polymerization. (**b**) SEM images showing the promixity effect, where the two zoom-in insets present the result with (top right) and without (bottom left) over-polymerization. Scale bars are 100 μm.

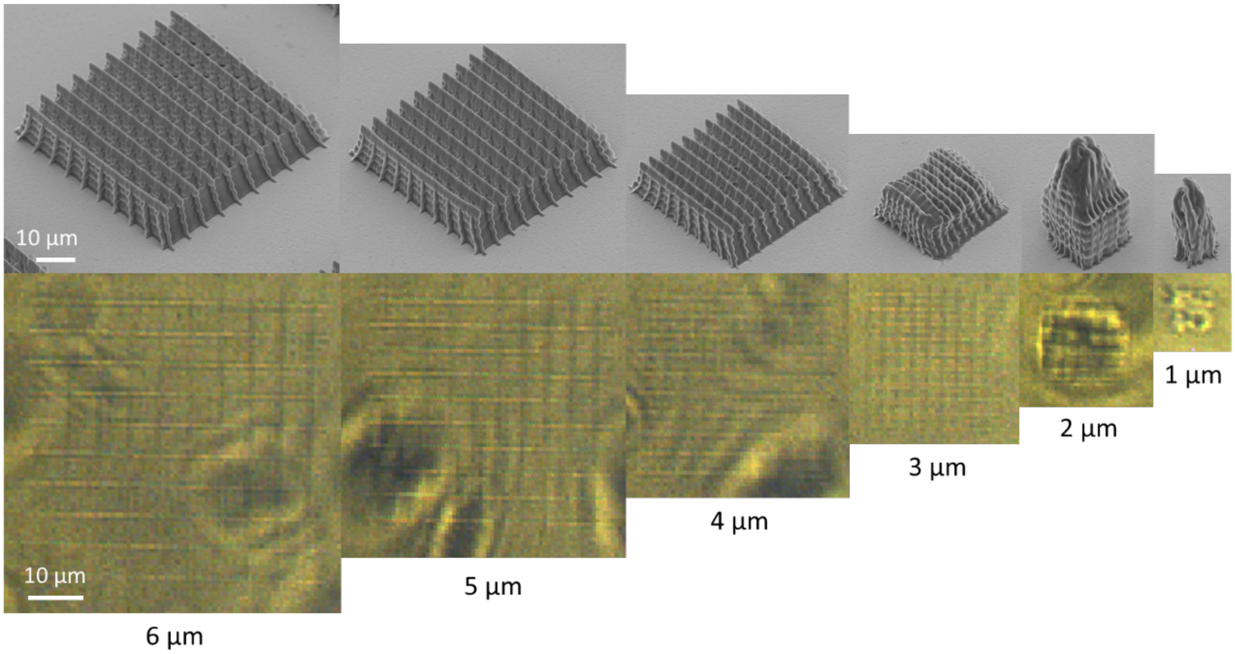

Supplementary Figure 9. SEM images (top) and in-situ printing optical images (bottom) of wood-pile structures printed via different focus distances from 6 to 1 µm. Scale bars are 10 µm. The effect of printing with different minimum focus distances (i.e., 6, 5, 4, 3, 2, and 1 µm) was investigated by printing woodpile structures using 100 foci. The results show that high quality structures without signs of over-polymerization can be achieved when the minimal focus distance is set to be larger than 3 µm. This result is consistent with the work of Arnoux, C. et al.[14]

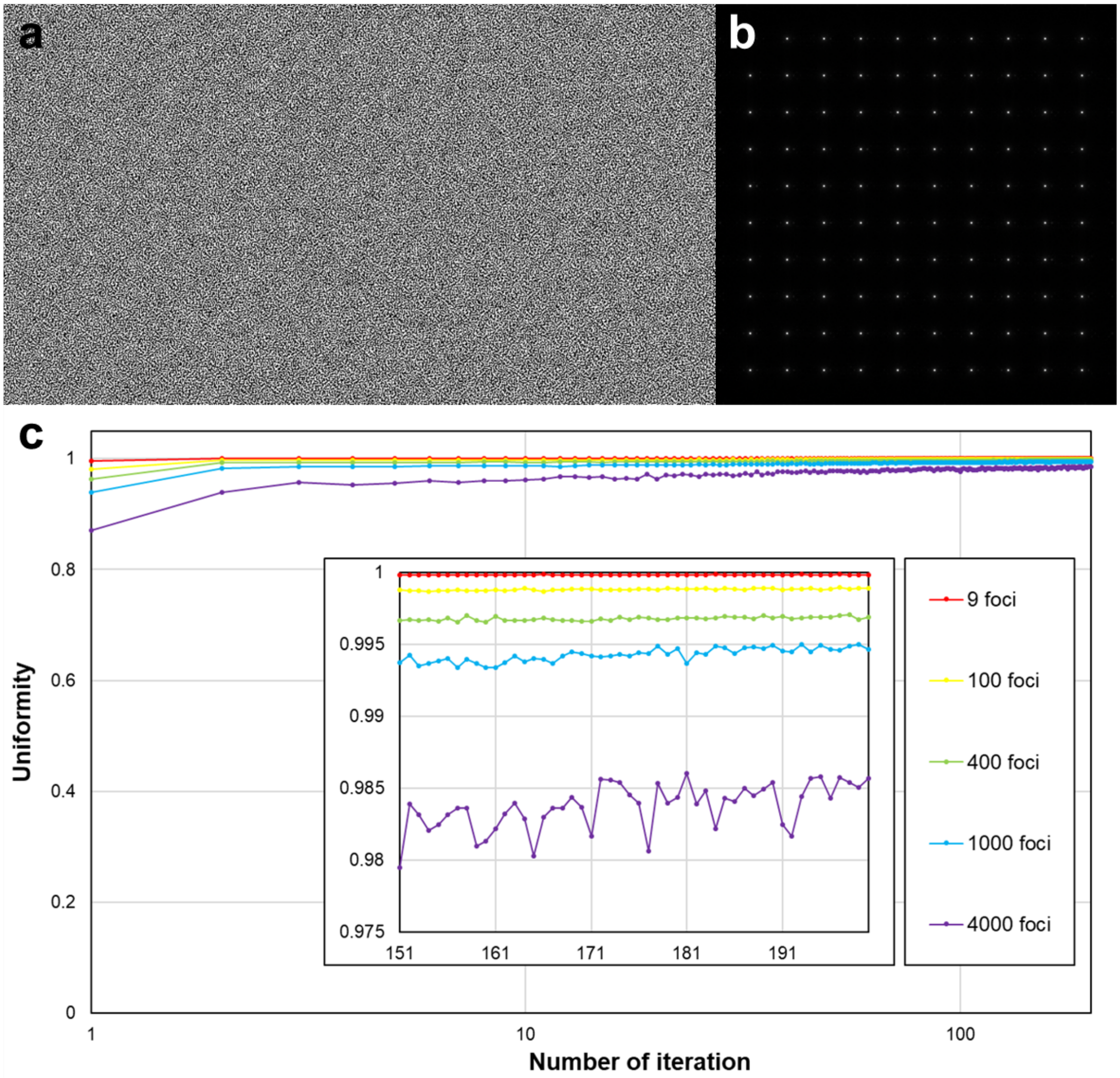

Supplementary Figure 10. (**a**) Example of an designed binary hologram containing 100 foci; and (**b**) simulated focus positions with equal intensity in the build plane. (**c**) Uniformity of laser intensity for 9, 100, 400, 1000, and 4000 foci plotted as a function of the number of iteration via the WGS algorithm. (Note that the horizontal axis is in logarithmic scale.)

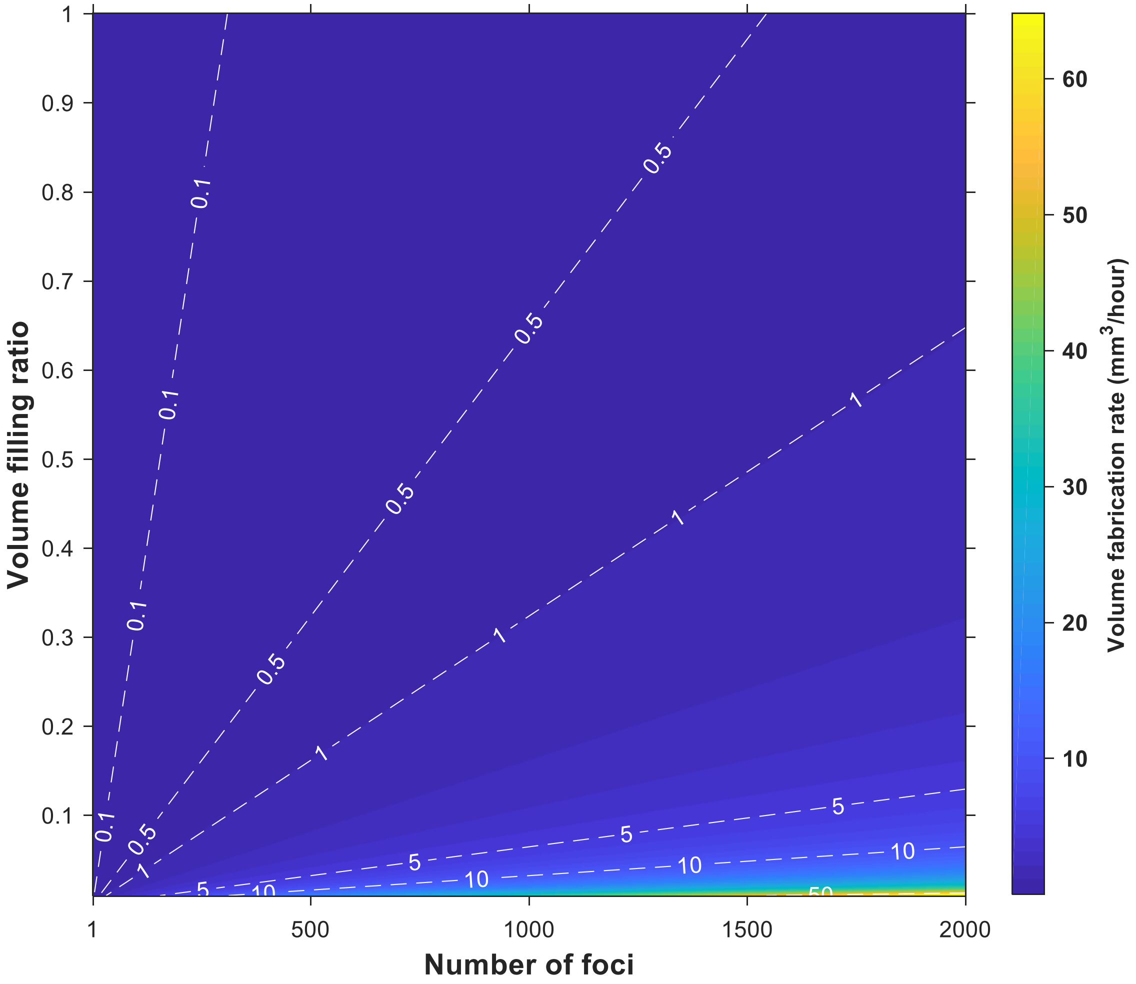

Supplementary Figure 11. Volume fabrication rate of the multi-focus TPL system plotted as a function of volume filling ratio and the number of foci in mm^3/hour.

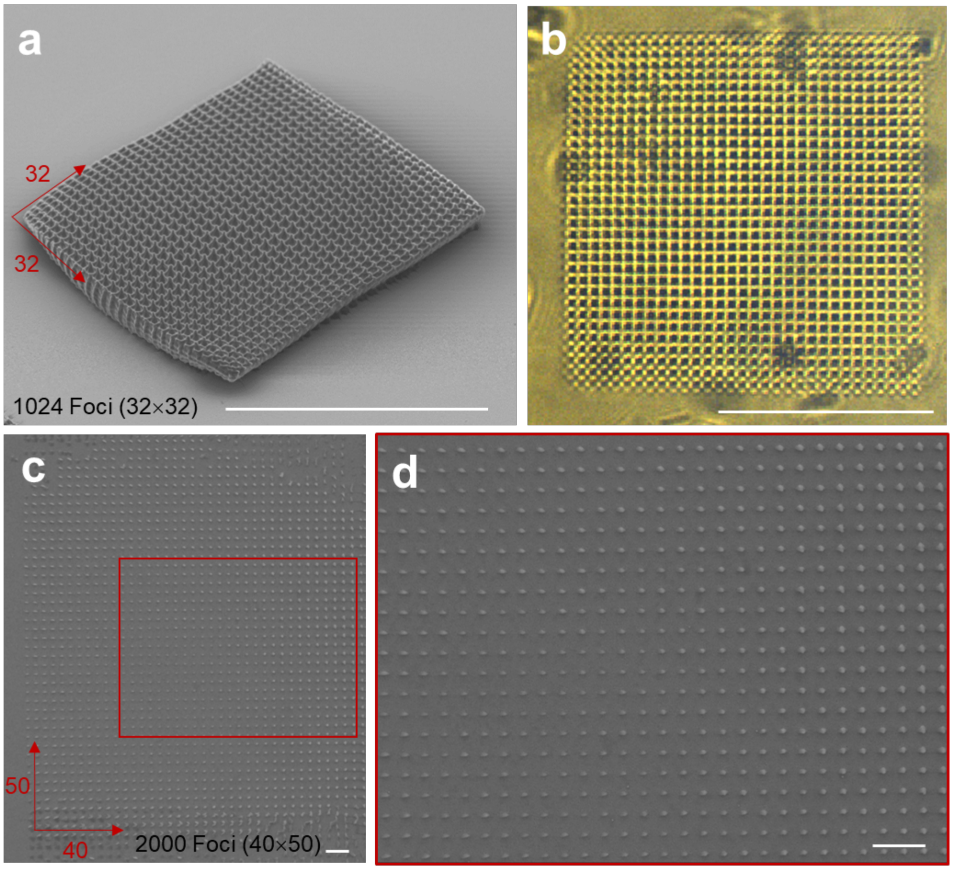

Supplementary Figure 12. Nanofabrication via the multi-focus TPL system: (**a**) SEM image of the octahedral struss structure fabricated by 1024 foci; (**b**) fabrication process of (a) recorded during printing. (**c**) SEM images of a nano-dot array printed by 2000 foci, and (**d**) zoom-in view of the red box in (c). Scale bars are 100 μm for (a) and (b); and 10 μm for (c) - (d). 1024 and 2000 laser foci were applied to parallely fabricate a octahedral struss structure and nano-dot array respectively. From Supplementary Fig. 12b, we may observe the structure was uniformly printed with high resolution. (The structure became distorted during the development process due to the structure internal stress.) The nano-dot array, which has a size of 160×200 μm^2, also demonstrates high uniformity and precision. These results confirm our method and system can achieve high printing quality using up to 2000 laser foci.

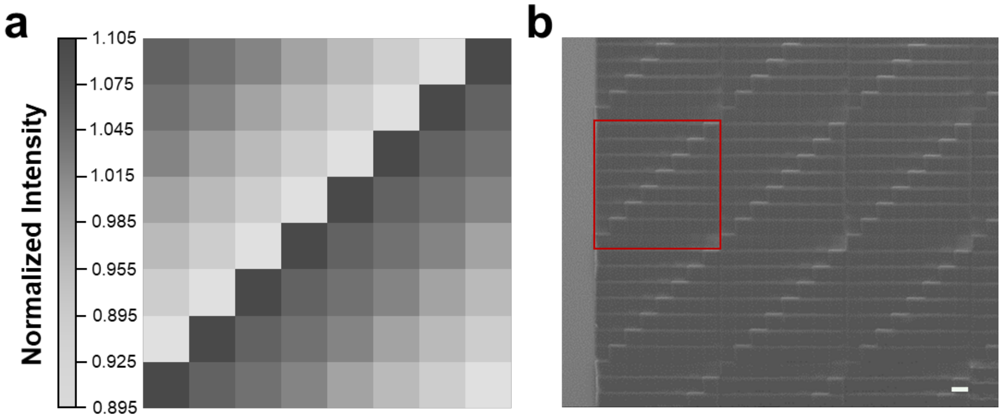

Supplementary Figure 13. (**a**) Design of a 2D grayscale checkerboard pattern with 8 different intensity levels, as shown in the scale bar. (**b**) SEM image of the fabricated checkerboard pattern using 64 foci. Scale bar is 10 μm in (b). This fabrication experiment demonstrates the grayscale printing capability of our method, i.e., each generated laser focus can carry different laser power with better than 99% accuracy. The power of each focus is independently adjusted by assigning the designed value to I_t^d in Eq. (11). The results shown in Supplementary Fig. 13b confirm the capability and accuracy of our method.

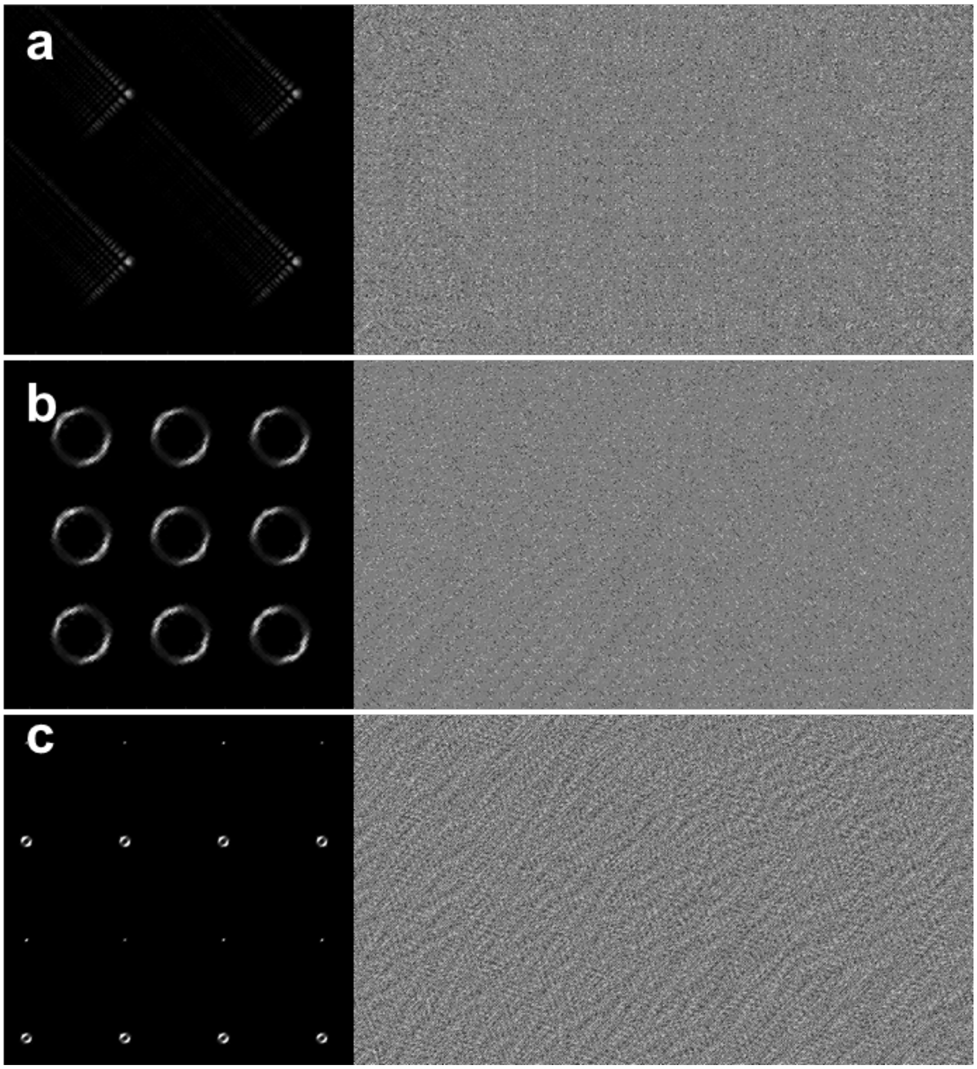

Supplementary Figure 14. Demonstration of multi-focus beam shaping: Generation of (**a**) four Airy beams, (**b**) nine Bessel beams and (**c**) eight Bessel beams as well as eight Gaussian beams simultaneously in MATLAB. The right colume shows the corresponding binary holograms. Multi-focus beam shaiping is realized by modulating the phase term $\phi(x, y)$ in Eq. (7). For example, the Airy beam and Bessel beam are generated by adding a phase equation of $\varphi_{Airy}(x,y) = 2\pi \cdot \frac{x^3+y^3}{\rho}$ and $\varphi_{Bessel}(x,y) = 2\pi \cdot \frac{\sqrt{x^2+y^2}}{\rho}$, respectively.

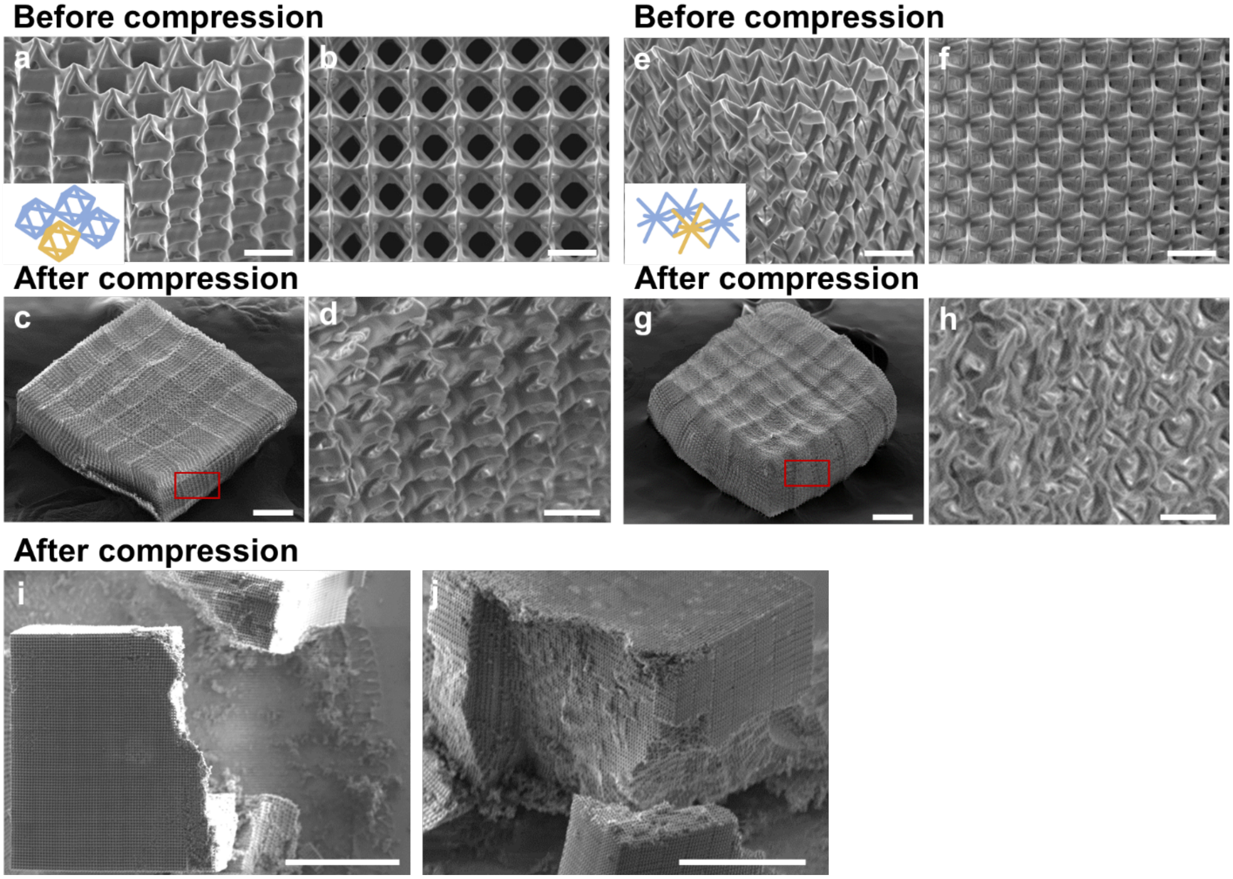

Supplementary Figure 15. Compression test of the fabricated metastructures: (**a**, **b**) Isometric and top views of a printed octahedral truss metastructure (Truss 1 in Fig. 3) before compression. (**c**) SEM image of the metastructure in (a) after compression (88% strain). (**d**) Zoom-in view of (c) in the red box. (**e**, **f**) Isometric and top views of a different truss metastructure (Truss 2 in Fig. 3) before compression. (**g**) SEM image of the metastructure in (e) after compression (88% strain). (**h**) Zoom-in view of (g) in the red box. (**i**, **j**) Top view and oblique view of a fractured carbonized metastructure (i.e., the structure in Fig. 3e) after the application of a 17% (strain) compression. Scale bars are: 10 µm for (a), (b), (d), (e), (f), and (h); and 100 µm for (c), (g), (i) and (j). The compression tests have verified the fabricated metastructures have the predicted high deformation characteristics. The results in Supplementary Fig. 15i-j show that carbonized metastructures have subtantially reduced plastic range (but with improved strength and stiffness as shown in Fig. 3f-g).

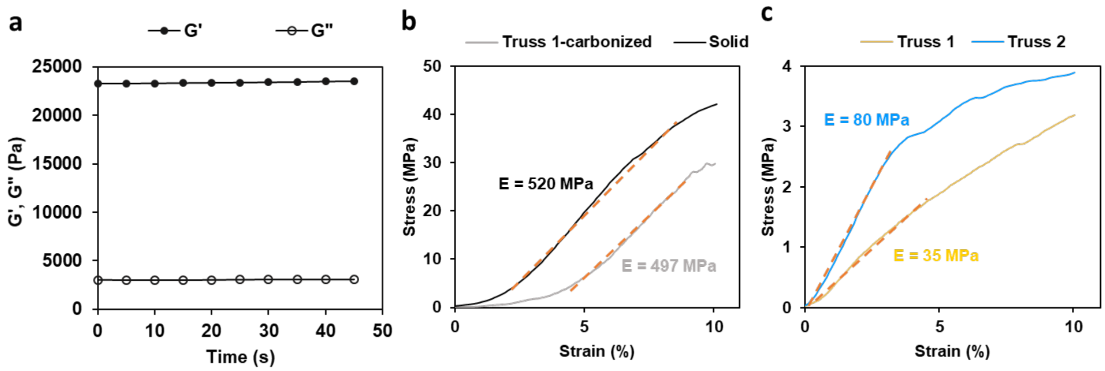

Supplementary Figure 16. Mechanical properties of the printed metastructures: (**a**) Measured storage modulus (*G'*) and loss modulus (*G''*) curves of a solid cube. (**b**) Measured stress-strain curve (from 0 – 10%) of the carbonized octahedral truss structure (gray line) in Fig. 3e and the solid cube (black line). (**c**) Measured stress-strain curve (from 0 – 10%) of Truss 1 (yellow line) and Truss 2 (blue line) in Fig. 3. The solid cube in Supplementary Fig. 16a was printed by 196 foci with a laser pulse energy of 5 nJ (per focus) and voxel distance of 300 nm and 1 μm in the lateral and axial directions, respectively. Truss 1 and 2 were printed by 100 foci with a laser pulse energy of 5 nJ (per focus) and a voxel distance of 150 nm and 1 μm in lateral and axial directions, respectively. Based on the results in Supplementary Fig. 16, the Young's modulus for the solid cube, carbonized Truss 1, Truss 1, and Truss 2 are calculated to be 520, 497, 35, and 80 MPa, respectively.

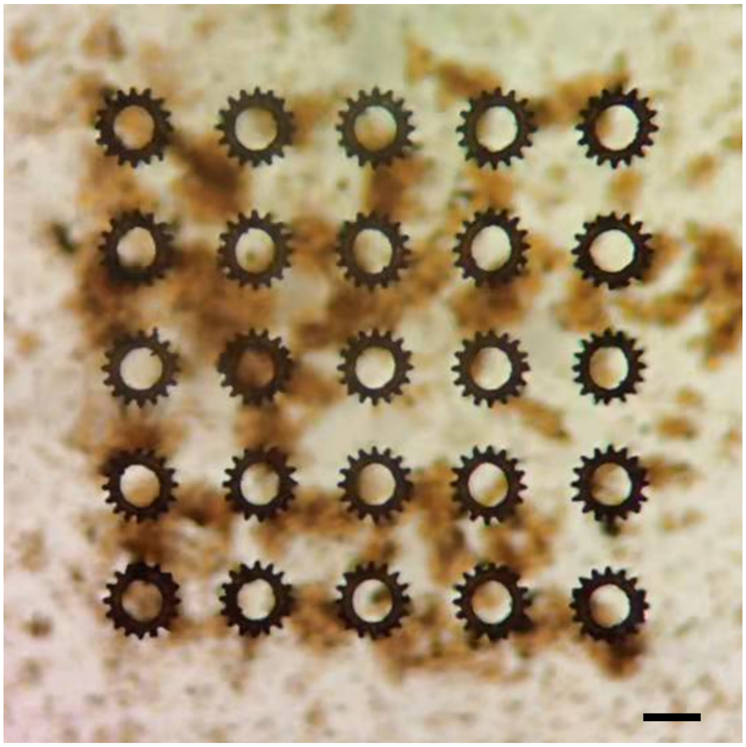

Supplementary Figure 17. Optical image of the magnetic micro-gear array in Fig. 4m. Scale bar is 50 µm.

Supplementary References

1. Moestopo, W. P., Mateos, A. J., Fuller, R. M., Greer, J. R., & Portela, C. M. Pushing and Pulling on Ropes: Hierarchical Woven Materials. *Adv. Sci.* **7,** 2001271 (2020).
2. Bauer, J., Kraus, J. A., Crook, C., Rimoli, J. J., & Valdevit, L. Tensegrity Metamaterials: Toward Failure-Resistant Engineering Systems through Delocalized Deformation. *Adv. Mater.* **33,** 2005647 (2021).
3. Crook, C. et al. Plate-nanolattices at the theoretical limit of stiffness and strength. *Nat. Commun.* **11,** 1-11 (2020).
4. Tancogne-Dejean, T., Diamantopoulou, M., Gorji, M. B., Bonatti, C., & Mohr, D. 3D Plate-Lattices: An Emerging Class of Low-Density Metamaterial Exhibiting Optimal Isotropic Stiffness. *Adv. Mater.* **30,** 1803334 (2018).
5. Saha, S.K. et al. Scalable submicrometer additive manufacturing. *Science* **366,** 105-109 (2019)
6. Lee, W.H. Binary synthetic holograms. *Appl. Opt.* **13,** 1677-1682 (1974).
7. Geng, Q., Wang, D., Chen, P. & Chen, S. C. Ultrafast multi-focus 3-D nano-fabrication based on two-photon polymerization. *Nat. Commun.* **10,** 1-7 (2019).
8. Geng, Q., Gu, C., Cheng, J. & Chen, S. Digital micromirror device-based two-photon microscopy for three-dimensional and random-access imaging. *Optica* **4,** 674 (2017).
9. Cheng, J., Gu, C., Zhang, D., Wang, D. & Chen, S.-C. Ultrafast axial scanning for two-photon microscopy via a digital micromirror device and binary holography. *Opt. Lett.* **41,** 1451–1454 (2016)
10. Cheng, J., Gu, C., Zhang, D. & Chen, S.-C. High-speed femtosecond laser beam shaping based on binary holography using a digital micromirror device. *Opt. Lett.* **40,** 4875–8 (2015).
11. Rekštytė, S., Jonavičius, T. & Malinauskas, M. Direct laser writing of microstructures on optically opaque and reflective surfaces. *Opt. Lasers Eng.* **53,** 90-97 (2014).
12. Baldacchini, T. *Three-dimensional Microfabrication Using Two-photon Polymerization: Fundamentals, Technology, and Applications Ch. 3* (William Andrew, Norwich, NY, 2015).
13. http://bigwww.epfl.ch/algorithms/psfgenerator/#psf
14. Arnoux, C. et al. Understanding and overcoming proximity effects in multi-spot two-photon direct laser writing. *Addit. Manuf.* 102491 (2021).
