# Supplementary material for: Ultrafast 3D nanofabrication via digital holography
Source: Nat Commun. 2023 Mar 27;14:1716. doi: 10.1038/s41467-023-37163-y (PMC10043265; doi:10.1038/s41467-023-37163-y)
Supplement: Supplementary file 3 — Description of Additional Supplementary Files [file 41467_2023_37163_MOESM3_ESM.pdf]

Description of Additional Supplementary Files:

Supplementary Movie 1: Parallel fabrication of an octahedral truss structure using 100 laser foci.

Supplementary Movie 2: Parallel fabrication of a 2D array of numbers and alphabets using 36 laser foci.

Supplementary Movie 3: Actuation of the printed micro-gear to generate the rotation motion at 5 Hz.

Supplementary Movie 4: Actuation of the printed micro-gear to generate the directional flipping motion at 1 Hz
